# Photon-Energy-Dependent Reversible Charge Transfer Dynamics of Double Perovskite Nanocrystal-Polymer Nanocomposites

**DOI:** 10.3390/nano12234300

**Published:** 2022-12-04

**Authors:** Ruixiang Wu, Xiaoshuai Wang, Jingjing Luo, Xin Liu, Fengjie Guo, Bin Li, Shengzhi Wang, Peigeng Han, Xiangyang Miao

**Affiliations:** 1 Key Laboratory of Spectral Measurement and Analysis of Shanxi Province, College of Physics and Information Engineering, Shanxi Normal University, Taiyuan 030031, China; 2 State Key Laboratory of Molecular Reaction Dynamics, Dalian Institute of Chemical Physics, Chinese Academy of Science, Dalian 116023, China; 3 Institute of Molecular Science and Engineering, Institute of Frontier and Interdisciplinary Science, Shandong University, Qingdao 266237, China

**Keywords:** transient absorption spectroscopy, reversible charge transfer, lead-free double perovskite, conjugated polymer, nanocomposites

## Abstract

Combining steady-state photoluminescence and transient absorption (TA) spectroscopy, we have investigated the photoinduced charge transfer dynamics between lead-free Mn-doped Cs_2_NaIn_0.75_Bi_0.25_Cl_6_ double perovskite (DP) nanocrystals (NCs) and conjugated poly[2-methoxy-5-(3′,7′-dimethyloctyloxy)-1,4-phenylenevinylene] (MDMO-PPV). Upon ultraviolet-A excitation, the photoinduced absorption feature of DP NCs/MDMO-PPV nanocomposites disappeared, and the stimulated emission weakened in the TA spectrum. This was due to charge transfer from the MDMO-PPV polymers to DP NCs. Upon a higher photon-energy ultraviolet-C excitation, stimulated emission and photoinduced absorption features vanished, indicating there existed a reversible charge transfer from DP NCs to MDMO-PPV polymers. Reversible charge transfer of Mn-doped DP NCs/MDMO-PPV nanocomposites was tuned by varying the excitation photon-energy. The manipulation of reversible charge transfer dynamics in the perovskite-polymer nanocomposites opens a new avenue for optical and optoelectronic applications.

## 1. Introduction

Perovskite nanocrystals (NCs) have the advantages of variable structures, large absorption coefficients, narrow emission bands, and high photoluminescence (PL) quantum yields [1,2,3,4]. This benefits light emitting diodes (LEDs) [5,6], photodetectors [7,8,9], sensors [10,11], and solar cells [12,13]. Researchers have focused most of their efforts on the development and characterization of perovskite NCs. There are some issues that limit their large-scale application, particularly in terms of conversion efficiency, spectral response range, and stability of the optoelectronic devices, which still need to be improved. Conjugated polymers possess mechanically flexible, unique intra- and inter-chain emission properties [14,15,16], and can provide efficient charge conduction to overcome the perovskite NCs’ weakness [17]. Recently, extensive works have been performed on perovskite NC-polymer composites, whose optical properties are effectively tuned to facilitate improved device performance [18,19]. Heo et al. had achieved an efficient photovoltaic performance in solar cells of three-dimensional composites of TiO_2_/CH_3_NH_3_PbI_3_ and complementary well matched polymeric hole conductors [19]. Future development and optimization of perovskite NC-polymer composites will require a deeper understanding of the critical processes, such as energy transfer and charge transfer [20,21,22,23]. Introducing CsPbBr_3_ NCs into poly[2-methoxy-5-(2-ethylhexyloxy)-1,4-phenylenevinylene] (MEH-PPV) and poly(9,9-di-*n*-octylfluorenyl-2,7-diyl) composite thin films improved the polymer blend crystallinities, reduced the localized densities of the electronic states, and enhanced energy transfers from the polymer blends to NCs [20]. The templating properties of MEH-PPV controlled the self-assembly of CH_3_NH_3_PbI_3_ NCs, and those nanocomposites possessed remarkable photovoltaic efficiencies due to charge transfer interface interactions [21]. In poly(9,9-dioctylfluorene-*co*-benzothiadiazole) and CsPbI_1.5_Br_1.5_ NCs hybrid film, charge transfer both from polymers to the NCs and from the NCs to polymers would take place, and this was expected to be relevant for the development of hybrid organic-perovskite optoelectronic devices [23].

Apart from Pb-based perovskite NCs, recently, environment-friendly lead-free double perovskite (DP) NCs with three-dimensional structure, nontoxicity, and stability have attracted wide attention and exhibit outstanding characteristics [24,25,26,27]. However, the interaction and charge carrier transfer dynamics between lead-free DP NCs and polymers are still unclear and remain to be studied. Here, we investigated the charge transfer dynamics of lead-free DP NC-polymer nanocomposites that comprised Mn-doped DP NCs and poly[2-methoxy-5-(3′,7′-dimethyloctyloxy)-1,4-phenylenevinylene] (MDMO-PPV, and other PPV related polymers). The PL emission and transient absorption (TA) spectra of DP NCs, MDMO-PPV, and DP NCs/MDMO-PPV nanocomposites in toluene were measured under different photon-energy excitation of ultraviolet-A (UVA) and ultraviolet-C (UVC) light. Reversible charge transfer dynamics were analyzed by combining PL and TA spectra and benefit light-harvesting systems.

## 2. Materials and Methods

MDMO-PPV (177716-59-5), cesium acetate (Cs(OAc), 99.99%), sodium acetate (Na(OAc), 99.99%, anhydrous), oleylamine (80%) were purchased from Aladdin (Shanghai, China). Indium acetate (In(OAc)_3_, 99.99%), manganese acetate (Mn(OAc)_2_, 98%, anhydrous), chlorotrimethylsilane (TMSCl, 98%), 1-octadecene (90%), and oleic acid (90%) were purchased from Alfa Aesar (MA, USA). Bismuth acetate (Bi(OAc)_3_, 99.99%) was purchased from Sigma-Aldrich (Steinheim, Germany). Toluene (99.5%) was purchased from Sinopharm Chemical Reagent Co., Ltd. (Shanghai, China).

Mn-doped Cs_2_NaIn_0.75_Bi_0.25_Cl_6_ DP NCs (with long PL lifetimes of ~8.7 ms [26]) were synthesized by a previously reported variable temperature hot injection [28,29,30,31]. 0.65 mmol Cs(OAc), 0.45 mmol Na(OAc), 0.325 mmol In(OAc)_3_, 0.125 mmol Bi(OAc)_3_, and 0.14 mmol Mn(OAc)_2_ were added in a mixture of oleic acid (2.8 mL), oleylamine (0.7 mL), and 1-octadecene (10 mL), which was heated to 110 °C under a vacuum for 60 min. The reaction mixture was heated through a temperature gradient of 6 °C/min under a nitrogen atmosphere, and TMSCl (0.4 mL) was swiftly injected at 168 °C. The reaction mixture continued to 180 °C and was immediately cooled to room temperature in an ice–water bath. The reaction mixture was then decanted into a centrifugal tube and centrifuged at 9,000 rpm for 20 min. The supernatant was removed. The precipitate was washed in 10 mL of toluene and centrifuged at 10,000 rpm for 15 min. The supernatant was discarded. The precipitate was redispersed in 5 mL of toluene with sonication and centrifuged at 6,000 rpm for 15 min, and colloidal Mn-Doped Cs_2_NaIn_0.75_Bi_0.25_Cl_6_ DP NCs were obtained by discarding the bottom precipitate. Mn-doped DP NCs and MDMO-PPV were stirred in toluene for 5 min by vortex mixer, and their concentration was 1 mg/mL and 0.04 mg/mL in DP NCs/MDMO-PPV nanocomposites, respectively.

The morphology of DP NCs, MDMO-PPV, and DP NCs/MDMO-PPV nanocomposites were characterized by transmission electron microscopy (TEM) by using the FEI Talos F200X (OR, USA). Absorption and PL emission spectra were acquired with a spectrofluorometer (Duetta, Horiba, Canada). TA spectroscopy was investigated by nanosecond laser flash photolysis (LFP-100, Dalian Institute of Chemical Physics, Liaoning, China). Harmonic pulse wavelengths [UVA (355 nm) and UVC (266 nm)] were generated using a Q-switched Nd:YAG laser (3 Hz, Nimma-900, Beamtech Optronics Corp.) as the pump source. Probe light was adopted to a Xenon lamp (XBO 450W/OFR, OSRAM), and the excited state dynamics of the DP NCs/MDMO-PPV nanocomposites were monitored from 280–700 nm. TA spectra were detected by a photomultiplier tube (CR131, Hamamatsu). In this experiment, the single pulse energy of the pump laser was ~30 mJ.

## 3. Results and Discussion

**Figure 1:**
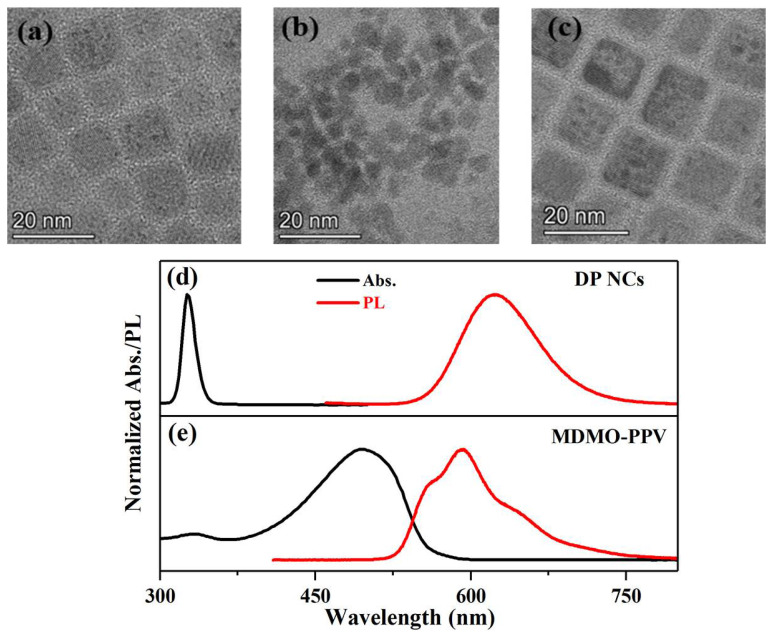
TEM images of (**a**) Mn-doped DP NCs, (**b**) MDMO-PPV polymers, and (**c**) DP NCs/MDMO-PPV nanocomposites. Normalized absorption (black line) and PL emission (red line) spectra of (**d**) Mn-doped DP NCs and (**e**) MDMO-PPV polymers in toluene.

TEM images of Mn-doped DP NCs, MDMO-PPV polymers, and DP NCs/MDMO-PPV nanocomposites are shown in Figure 1a–c. TEM images show evenly distributed cubic-shaped Mn-doped DP NCs with a mean size of ~10.5 nm (Figure 1a). The average size of the MDMO-PPV nanoparticles was ~4.3 nm (Figure 1b). Conjugated polymer nanoparticles would be easily to aggregate together. In the TEM image of DP NCs/MDMO-PPV nanocomposites (Figure 1c), it was suggested that MDMO nanoparticles were more inclined to adhere to the surface of DP NCs. DP NCs/MDMO-PPV nanocomposites had hybrid nanostructures.

Absorption and PL emission spectra of DP NCs and polymers are shown in Figure 1d,e. Mn-doped DP NCs show a narrow absorption at 335 nm and emit a red PL at 624 nm attributed to the spin forbidden nature of the Mn^2+^ dopants (^4^T_1_ → ^6^A_1_ transition) (Figure 1d). MDMO-PPV polymers in toluene displayed a broad absorption band with a peak at 496 nm (Figure 1e) due to strong π−π* transitions [32]. The corresponding PL band maximum occurred at 590 nm with a shoulder ~640 nm. 

**Figure 2:**
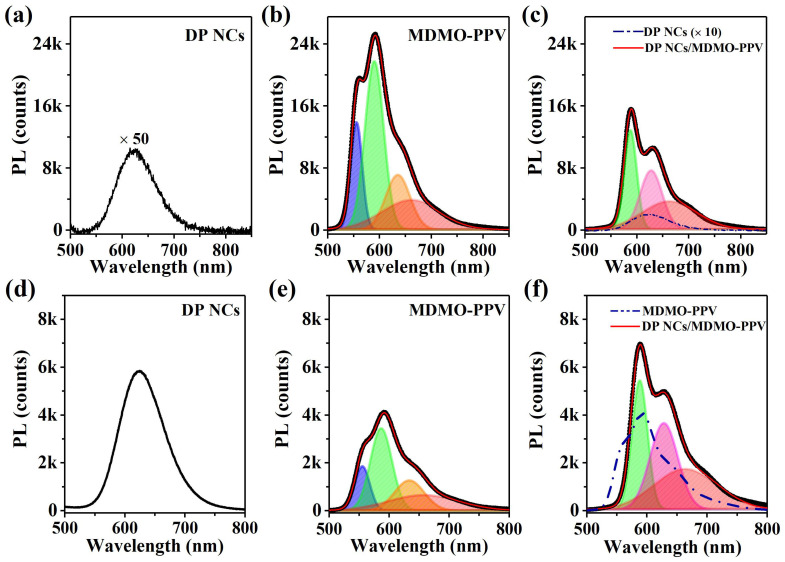
(**a**) PL emission spectrum of DP NCs and (**b**) PL emission spectra (black point) of MDMO-PPV polymers in toluene with their corresponding four-peak Gaussian fits at 555 nm (blue), 590 nm (green), 635 nm (orange), and 660 nm (red), as well as (**c**) PL emission spectra of DP NCs/MDMO-PPV nanocomposites and their corresponding three-peak fitting at 590 nm (green), 628 nm (pink), and 660 nm (red) under UVA excitation. (**d**) PL spectrum of DP NCs and (**e**) PL emission spectra (black point) of MDMO-PPV polymers in toluene with their corresponding four-peak Gaussian fits at 555 nm (blue), 590 nm (green), 635 nm (orange), and 660 nm (red), as well as (**f**) PL emission spectra of DP NCs/MDMO-PPV nanocomposites and their corresponding three-peak fitting at 590 nm (green), 628 nm (pink), and 660 nm (red) under UVC excitation.

Figure 2 shows PL spectra of Mn-doped DP NCs, MDMO-PPV, and DP NCs/MDMO-PPV nanocomposites excited at UVA (355 nm) and UVC (266 nm). The PL spectrum of DP NCs/MDMO-PPV nanocomposites changed. DP NCs presented a very weak PL merely in Figure 2a, because it is difficult to be excited by UVA excitation. The PL emission spectrum of MDMO-PPV polymers was fit using a four-peak Gaussian function, as shown in Figure 2b. They corresponded to the intra-chain exciton emission at 555 nm and inter-chain exciton emissions at 590 nm, 635 nm, and 660 nm [31,32,33]. As shown in Figure 2c, introducing the DP NCs into the MDMO-PPV solution caused the intra-chain exciton PL emission at 555 nm to disappear due to MDMO-PPV chain folding [31] and suppression of the intra-chain emission. For the inter-chain exciton emission of MDMO-PPV, DP NCs/MDMO-PPV nanocomposites exhibited the quenched PL at 590 nm and enhanced PL of DP NCs at 624 nm. Upon a higher photon-energy UVC excitation, PL emissions of DP NCs/MDMO-PPV nanocomposites at 590 nm and 660 nm increased, as seen in Figure 2f, compared to MDMO-PPV polymers. These spectral changes indicated an interaction between Mn-doped DP NCs and MDMO-PPV polymers in the nanocomposites.

**Figure 3:**
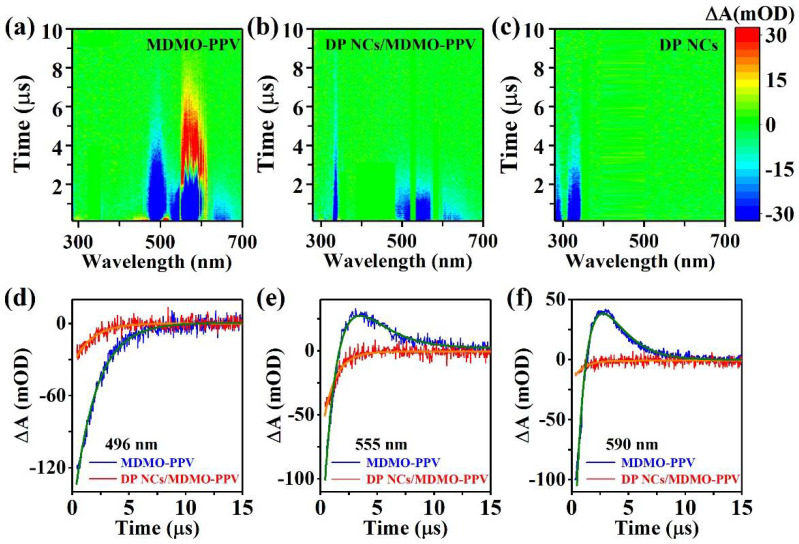
Transient absorption (TA) spectra at UVA excitation in toluene of (**a**) MDMO-PPV polymers, (**b**) DP NCs/MDMO-PPV nanocomposites, and (**c**) Mn-doped DP NCs, respectively. TA dynamic trajectories and their corresponding fitting curves for MDMO-PPV polymers (blue line) and DP NCs/MDMO-PPV nanocomposites (red line) at probe wavelengths of (**d**) 496 nm, (**e**) 555 nm, and (**f**) 590 nm, respectively.

Nanosecond TA measurements were conducted to investigate the excited-state interaction between Mn-doped DP NCs and MDMO-PPV polymers. Upon UVA pulse laser excitation, TA plots of MDMO-PPV polymers in Figure 3a contain a ground state bleach (GSB, ΔA < 0, 470–520 nm), a stimulated emission (SE, ΔA < 0, 520–680 nm), and photoinduced absorption (PIA, ΔA > 0, 550–610 nm) features. The spectral range of negative polymer GSB and SE features in the TA plots match their steady-state absorption band at 496 nm and PL emission, respectively. In the presence of DP NCs, the PIA feature of MDMO-PPV polymers disappears at 550–610 nm (Figure 3b). The GSB feature from 470–520 nm weakened, with an emergent TA signal at 335 nm, which originated from the GSB feature of Mn-doped DP NCs, as illustrated in Figure 3c. The SE feature of DP NCs/MDMO-PPV nanocomposites (600–650 nm) blue shifted relative to MDMO-PPV polymers at 630–680 nm due to the electric field produced by excess electrons on the DP NC surfaces, which elevated the lowest excited state of the DP NCs [34,35]. We investigated the TA dynamic trajectories at different probe wavelengths to understand the revolution in the time domain. The 496 nm absorption band of MDMO-PPV shows a strong GSB feature, and TA dynamics obey single exponential decay with a lifetime of $\tau_{\mathrm{GSB}}^{\mathrm{MDMO}-\mathrm{PPV}}=2.00\;\mu s$ (Figure 3d). In the presence of DP NCs, the GSB features of MDMO-PPV polymers decreased dramatically. The $\tau_{\mathrm{GSB}}^{\mathrm{DP}\;\mathrm{NCs}/\mathrm{MDMO}-\mathrm{PPV}}=1.58\;\mu s$ became shorter for DP NCs/MDMO-PPV nanocomposites. Figure 3e,f show the TA dynamic trajectories at 555 nm and 590 nm, which correspond to the intra- and inter-chain emission of MDMO-PPV polymers, respectively. Both negative SE and positive PIA features of polymers were observed. The negative single recovered to 0, increased to its maximum positive value, then gradually decayed to 0. The trajectory was well fit using the following two-exponent decay formula:

(1) $$f\left(t\right)=f_{0}+p_{1}\exp\left(-t/\tau_{\mathrm{PIA}}\right)-p_{2}\exp\left(-t/\tau_{\mathrm{SE}}\right)$$

where $p_{1}$, $p_{2}$ > 0, $\tau_{\mathrm{PIA}}$, and $\tau_{\mathrm{SE}}$ correspond to their lifetimes. The fitting lifetimes are listed in Table 1, and $\tau_{\mathrm{PIA}}>\tau_{\mathrm{SE}}$ for MDMO-PPV polymers. In DP NCs/MDMO-PPV nanocomposites, the PIA feature disappeared, and the SE feature changed faster, along with a shorter lifetime. In addition, the SE feature has also been grasped from TA dynamic trajectories of MDMO-PPV and DP NCs/MDMO-PPV nanocomposites at 635 nm and 660 nm (other PL peaks of MDMO-PPV). This suggests that, when the nanocomposites are excited with UVA laser, the photo-induced charge carriers generated on the polymers will be rapidly transferred to the DP NCs. Therefore, the emission of the polymer can be suppressed, while the emission of the DP NCs can be enhanced.

**Figure 4:**
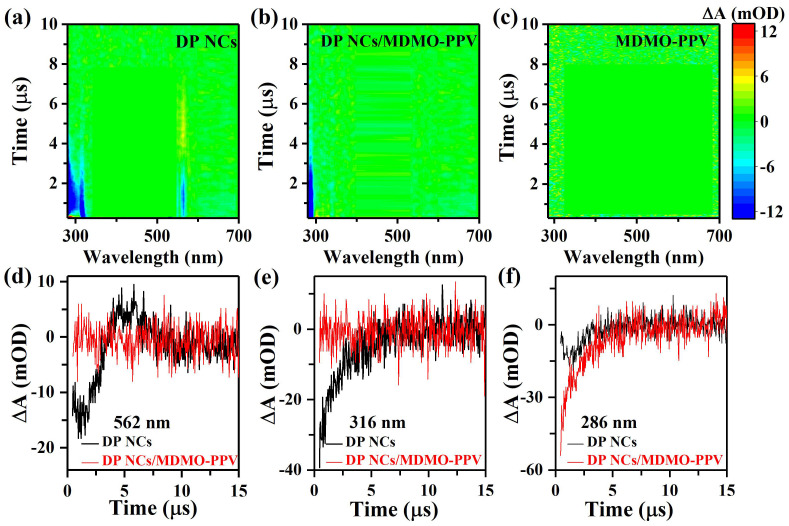
TA plots of (**a**) Mn-doped DP NCs, (**b**) DP NCs/MDMO-PPV nanocomposites, and (**c**) MDMO-PPV polymers. TA dynamic trajectories of DP NCs and DP NCs/MDMO-PPV nanocomposites at probe wavelengths of (**d**) 562 nm, (**e**) 316 nm, and (**f**) 286 nm. All measurements were excited by UVC laser.

For UVC pulse laser excitation, the TA spectrum of Mn-doped DP NCs consists of a broad GSB feature (280–320 nm) and weak SE and PIA features (560–565 nm) (Figure 4a). For both UVA and UVC excitations, DP NCs exhibit similar GSB features. SE occurs upon excitation at UVC due to the excitation difficulty at UVA for DP NCs. The TA spectrum of the nanocomposites changed (Figure 4b). SE and PIA features at 562 nm disappeared, and the GSB feature of DP NCs decreased in the presence of MDMO-PPV polymers (Figure 4d). For MDMO-PPV, there is no TA signal in Figure 4c, consistent with a weaker PL emission. In the DP NCs/MDMO-PPV nanocomposites, similar TA recovery curves occur at 286 nm, similar to DP NCs at 316 nm (Figure 4e,f), which indicated that they came from the GSB feature of DP NCs.

These results revealed an excited-state interaction between Mn-doped DP NCs and MDMO-PPV polymers in the nanocomposites. It can be found from Figure 1d,e that there is no spectral overlap between the absorption of DP NCs and the PL emission of MDMO-PPV, or between the PL emission of DP NCs and the absorption of MDMO-PPV. It indicates that charge transfer, rather than Förster resonance energy transfer, occurs in the nanocomposites [36]. On the one hand, by absorbing a pump photon energy in the UVA region (355 nm), the electron in the HOMO of MDMO-PPV polymers is excited to the LUMO. The LUMO electron transitions again upon absorbing another probe photon, or the downward transition to its HOMO along with emitting photon [37,38,39]. Those two electronic transitions explain the PIA feature at 550–610 nm and the SE feature at 520–680 nm, respectively. With the non-PL DP NCs introduced into MDMO-PPV, the PIA feature of polymers disappears in the TA spectrum. This indicated suppression of upward electronic transition, and LUMO electrons tended to transfer into the electron-acceptor DP NCs, which resulted in a stronger red PL emission [40]. The faster SE feature of DP NCs/MDMO-PPV nanocomposites implied a charge transfer from MDMO-PPV to the DP NCs. On the other hand, upon a higher photon-energy UVA (266 nm) excitation, Mn-doped DP NCs presented the PIA and SE features at 560–565 nm, which would disappear in the presence of MDMO-PPV polymers for the nanocomposites. The results indicated charge transfer direction in DP NCs/MDMO-PPV nanocomposites was reversed [23,41]. The reversible charge transfer from Mn-doped DP NCs to MDMO-PPV polymers in the nanocomposites generated the stronger PL of MDMO-PPV polymers.

## 4. Conclusions

In conclusion, we investigated the excited-state interactions in Mn-doped DP NCs/MDMO-PPV nanocomposites by steady-state and transient measurements. Excitation at a low photo-energy UVA region suppressed the upward electronic transition from the LUMO of MDMO-PPV polymers. Charge transfer from the polymers to Mn-doped DP NCs would produce the disappearance of the PIA polymer feature in the TA spectrum and would quench PL of MDMO-PPV. When DP NCs/MDMO-PPV nanocomposites were excited at a higher photon-energy UVC region, reversible charge transfer from DP NCs to the polymers occurred, which caused the disappearance of PIA and SE features and boosted the features of PL of MDMO-PPV polymers. Excitation-energy-dependent reversible charge transfer dynamics in the nanocomposites provide new possibilities for improving the performance of optoelectronic devices such as solar cells.

## Figures and Tables

**Table 1:** Fitting parameters of TA trajectories in Figure 3d–f.

λ (nm)	MDMO-PPV (µs)	DP NCs/MDMO-PPV (µs)
496	τ_GSB_ = 2.00	τ_GSB_ = 1.58
555	τ_PIA_ = 1.30, τ_SE_ = 1.22	τ_SE_ = 1.08
590	τ_PIA_ = 1.48, τ_SE_ = 1.45	τ_SE_ = 0.92
635/660	τ_SE_ = 1.32	τ_SE_ = 0.94

## Data Availability

All data generated and analyzed during this study are included in this published article.
